# Development of an electronic patient-reported outcome measure (ePROM) system to aid the management of patients with advanced chronic kidney disease

**DOI:** 10.1186/s41687-020-00223-8

**Published:** 2020-07-08

**Authors:** Derek Kyte, Nicola Anderson, Ram Auti, Olalekan Lee Aiyegbusi, Jon Bishop, Andrew Bissell, Elizabeth Brettell, Melanie Calvert, Marie Chadburn, Paul Cockwell, Mary Dutton, Helen Eddington, Elliot Forster, Gabby Hadley, Natalie J. Ives, Louise Jackson, Sonja O’Brien, Gary Price, Keeley Sharpe, Stephanie Stringer, Gael Stephenson, Rav Verdi, Judi Waters, Adrian Wilcockson, Jim Williams

**Affiliations:** 1 Centre for Patient-Reported Outcomes Research, University of Birmingham, Birmingham, UK; 2 Institute of Applied Health Research, University of Birmingham, Birmingham, UK; 3 National Institute for Health Research Birmingham Biomedical Research Centre, The Murray Learning Centre, University of Birmingham, Birmingham, UK; 4 University Hospitals Birmingham NHS Foundation Trust, Birmingham, UK; 5 Birmingham Clinical Trials Unit (BCTU), Institute of Applied Health Research University of Birmingham, Birmingham, UK; 6 Patient Advisory Group, Centre for Patient-Reported Outcomes Research, Institute of Applied Health Research, University of Birmingham, Birmingham, UK; 7 National Institute for Health Research Applied Research Centre West Midlands, University of Birmingham, Birmingham, UK; 8 National Institute for Health Research Surgical Reconstruction and Microbiology Research Centre, University of Birmingham, Birmingham, UK; 9 Birmingham Health Partners Centre for Regulatory Science and Innovation, University of Birmingham, Birmingham, UK; 10 Health Economics Unit, Institute of Applied Health Research, University of Birmingham, Birmingham, UK

**Keywords:** Chronic kidney disease, Patient-reported outcomes, Symptom monitoring

## Abstract

**Background:**

Effective management of patients with chronic kidney disease (CKD) relies on timely detection of clinical deterioration towards end stage kidney failure. We aimed to design an electronic Patient-Reported Outcome Measure (ePROM) system, which would allow patients with advanced CKD (pre-dialysis) to: (i) remotely self-report their symptoms using a simple and secure online platform; (ii) share the data with the clinical team in real-time via the electronic patient record to help optimise care. We adopted a staged development process which included: a systematic review of PROMs used in CKD; formation of a co-design team; prototype system design/development, user acceptance testing and refinement; finalisation of the system for testing in a pilot/feasibility trial.

**Results:**

A co-design team was convened, including patients with lived experience of CKD; clinical team members; IT/Informatics experts; academics; and Birmingham Clinical Trials Unit representatives. A prototype system was developed and iterative changes made before finalisation during a series of operational meetings. The system allows patients to remotely self-report their symptoms; provides tailored self-management advice; allows monitoring of real-time patient ePROM data; sends automated notifications to the patient/clinical team in the advent of a severe symptom report; and incorporates longitudinal ePROM symptom data into the electronic patient record. Feasibility of the system will be evaluated as part of the National Institute for Health Research funded RePROM (Renal electronic Patient-Reported Outcome Measure) pilot trial (ISRCTN12669006).

**Conclusions:**

Routine ePROM collection with real-time feedback has the potential to improve outcomes and reduce health service costs. We have successfully developed a trial-ready ePROM system for advanced CKD, the feasibility of which is currently being explored in a pilot trial. Assuming feasibility is demonstrated, formal evaluation of efficacy will take place in a future multi-centre randomised controlled trial.

## Background

The global burden of chronic kidney disease (CKD) continues to increase, with age-standardised death and disability-adjusted life years rising over recent decades [1]. Patients with advanced CKD (pre-dialysis) commonly experience a high symptom burden and impaired health-related quality of life, which represents a significant driver of outcomes and can be a particular source of anxiety [2–4].

With increasing use of digital healthcare, there has been much interest in the potential of harnessing electronic Patient-Reported Outcome Measures (ePROMs) to aid the management of symptom burden and optimise use of limited healthcare resources [5]. These measures allow patients to self-report their individual symptoms and overall symptom burden remotely using online platforms, with the opportunity to make the arising data available to health professionals in real-time to help support care [6].

Evidence in an oncology setting suggests patients are willing to complete ePROM symptom questionnaires on a regular basis, and that the data can be integrated into the electronic patient record (EPR), with beneficial results. Studies in cancer populations suggest that ePROM symptom monitoring may be associated with enhanced patient-clinician communication and patient activation; earlier detection of adverse events; improved patient quality of life; reduced use of accident and emergency services; fewer inpatient hospital episodes; and improved survival, even for ‘computer-inexperienced’ patients [7–14].

Whilst disease-specific PROMs are commonly used in pre-dialysis renal research, for example the Kidney Disease Quality of Life—36 (KDQOL- 36) and KDQOL-SF measures [15, 16], there has been relatively little research exploring the role of ePROMs in online renal symptom monitoring. The feasibility of routine ePROM capture has been demonstrated in patients with end stage kidney disease on home or in-centre dialysis, and in patients with CKD stage 4/5 (pre-dialysis), but without real-time feedback of data [17, 18]. A multi-centre randomised controlled trial (RCT) is therefore required to evaluate ePROM use with real-time feedback and EPR data integration to determine if health professionals, providers and policy-makers should implement systems in routine clinical practice. Before a definitive trial is undertaken, the Renal electronic Patient-Reported Outcome (RePROM) pilot trial is being conducted to assess feasibility and determine the key design elements for the full-scale RCT. Here, we describe the development of the RePROM intervention: an ePROM system for symptom monitoring in patients with advanced CKD (pre-dialysis).

## Methods

Development of the RePROM symptom reporting system is reported according to the Criteria for Reporting the Development and Evaluation of Complex Interventions in healthcare: revised guideline (CReDECI 2) [19].

### Setting

The RePROM study was undertaken within the Centre for Patient-Reported Outcomes Research at the University of Birmingham and the Queen Elizabeth Hospital Birmingham (QEHB) within the UK National Health Service (NHS) University Hospitals Birmingham Foundation Trust (UHBFT).

### Patient and public involvement

Development of the study design was informed by a series of meetings held with the study Patient Advisory Group (PAG), which included people with lived experience of CKD. PAG members were recruited via a targeted invitation distributed by renal staff at QEHB and academic staff within the Centre for Patient-Reported Outcome Research (CPROR) at the University of Birmingham. Members contributed to the design of the project prior to the application for funding, and the subsequent refinement of the protocol and patient-facing documentation, and were also involved in the ePROM co-design group, as outlined below. The PAG received compensation for their involvement in line with guidelines provided by the National Institute for Health Research INVOLVE UK national advisory group (https://www.invo.org.uk).

### Underlying theoretical basis

Development of the ePROM system drew upon underpinning theory around the use of such data for the purposes of clinical monitoring and the promotion of enhanced patient-centred care, self-management and clinical communication [20]. In particular, the theoretical models of Greenhalgh [20–22] and others [23–26]. Which highlight the potential of PROMs as a tool to support clinical management of patients, where patient-centred instruments are used to continually feedback data to all individuals involved in the care of the patient, in a format that allows integration within existing clinical data. We also sought to build a platform that might conform to models describing the role of PROM data in supporting: patient-clinician communication; shared understanding and decision-making; and optimal patient self-management of symptoms [20, 27, 28].

The core co-design group set a four-fold remit for the system:

1. To allow patients with advanced CKD to remotely self-report their symptoms using a simple and secure online platform.
2. To provide appropriate self-management advice to patients whose ePROM scores highlighted one or more mild/moderate/severe symptoms.
3. To allow monitoring of real-time patient ePROM symptom data and subsequent automated notification of both the patient and the clinical team in the advent of a severe symptom report.
4. To incorporate longitudinal ePROM symptom data in the EPR to help inform clinical consultations and foster shared understanding/decision-making.

### Design components

A staged development process was adopted, which included: a systematic review of PROMs used in CKD; formation of a co-design team; prototype system design/development, user acceptance testing and refinement; and finalisation of the system for use in the RePROM pilot/feasibility trial.

A systematic review of the literature, reported elsewhere [29], was undertaken to explore the measurement properties of PROMs used in adult patients with CKD and inform development of the ePROM system. Screening of 3702 titles/abstracts yielded 66 papers meeting the eligibility criteria, which described 25 PROMs. Three disease-specific PROMs were used in pre-dialysis populations: Agarwal [30], KDQOL- 36 [31, 32] and KDQOL-SF [33, 34]. There was evidence to support some satisfactory measurement properties (Agarwal: limited evidence for test-retest reliability and content validity; KDQOL-SF: moderate evidence for test-retest reliability and hypothesis testing; KDQOL-36: strong evidence for internal consistency, moderate evidence for hypothesis testing). However, these tools were missing evidence to support many other important properties (Agarwal: internal consistency, measurement error, structural validity, hypothesis testing, responsiveness; KDQOL-SF: internal consistency, measurement error, content validity, structural validity, responsiveness; KDQOL-36: reliability, measurement error, content validity, structural validity, responsiveness). In addition, none of the measures had been validated in a UK pre-dialysis population.

In parallel, a co-design team was convened, comprising: patients with lived experience of CKD (*n* = 6) and cancer (*n* = 1), who were members of the study patient advisory group; members of the broader renal clinical team at QEHB (*n* = 7), UHBFT IT/Informatics experts (*n* = 3); academics (*n* = 3); and members of the Birmingham Clinical Trials Unit (BCTU) (*n* = 5). In addition, design input was sought from international ePROM experts Profs Ethan Basch (University of North Carolina, United States), Niels Hjöllund (Arhuus University, Denmark) and Galina Velikova (Patient Outcomes Group, University of Leeds, United Kingdom).

User acceptance testing of a prototype version of the system, described in detail elsewhere [35], was conducted with 8 adult patients with advanced CKD (stages 4/5); the average usability and satisfaction score was 4.6 (5-point scale). Iterative changes were made to the system in response to the findings as outlined below.

Our initial intention was to incorporate an existing, validated, PROM within the final system. However, as outlined above, the systematic review failed to identify a valid, reliable, responsive and non-burdensome measure suitable for symptom monitoring in a UK pre-dialysis population [29]. The PAG and clinical members of the co-design group were therefore unable to select an existing validated measure which captured information on all identified patient- and clinician-important symptoms, without potentially burdening end- users with multiple questionnaires containing duplicate items. In order to proceed with feasibility testing of the ePROM system, we therefore developed stand-alone symptom-based questionnaire items based on the core actionable symptoms identified by our patient advisory group members and the UHBFT renal clinical team. In addition, the format and presentation of both the questions and response options was altered to increase font size and aid readability. Most questions were also allocated to a single page of the questionnaire to enhance clarity. A flag was added to highlight unanswered mandatory questions and a progress bar inserted as a point of reference for questionnaire completion to facilitate user orientation. Design of the ePROM system was finalised during a series of operational meetings held with the co-design team in 2017–2018.

## Results

### System architecture

**Fig. 1:**
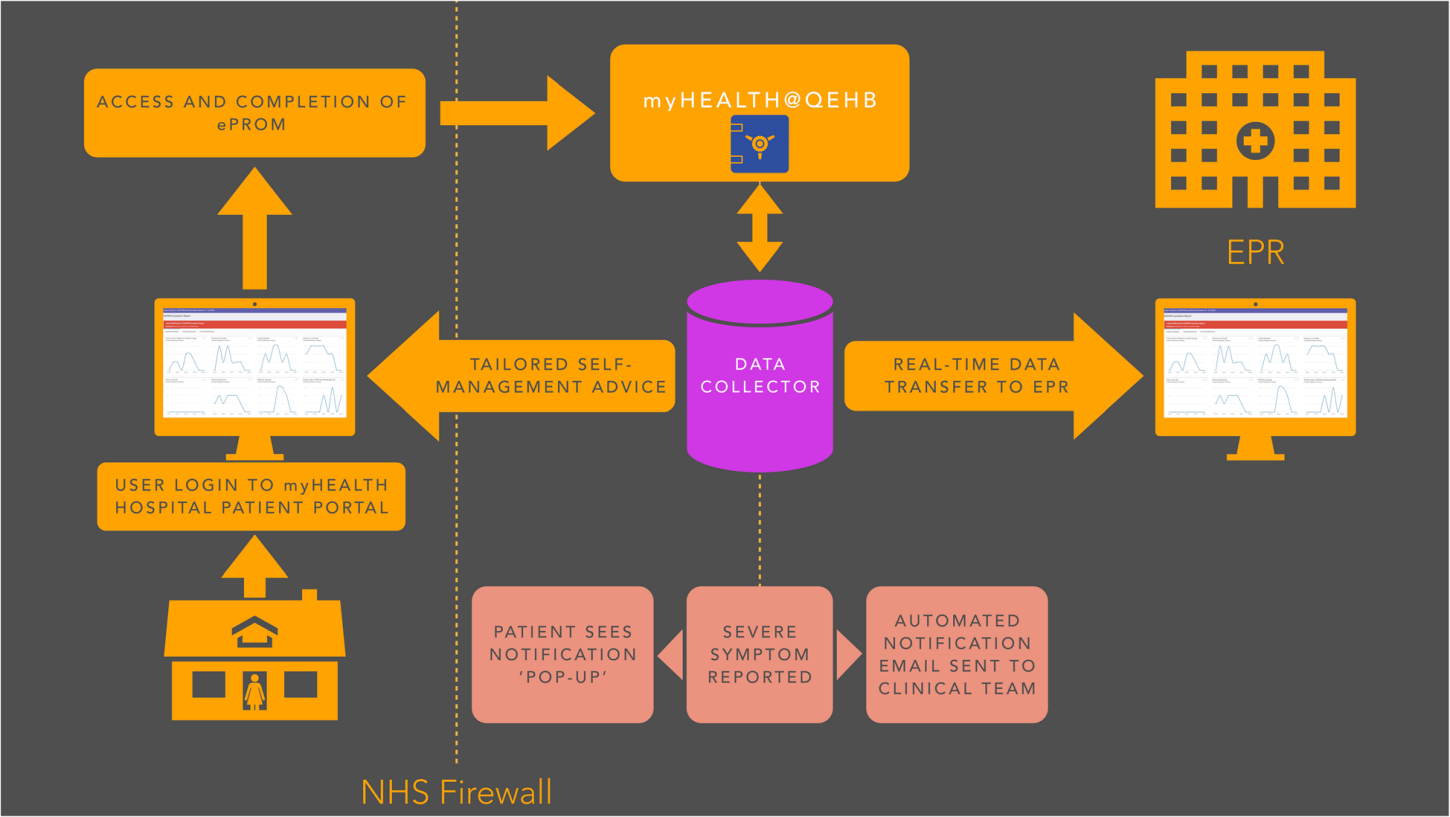
Overview of RePROM system architecture

The final system architecture is shown in Fig. 1. Patients access the ePROM system via the existing QEHB patient portal ‘myHealth’, which sits behind the NHS firewall. Approximately 20,000 patients with various conditions who receive care at QEHB are signed up to myHealth. These patients are able to use myHealth to access outpatient appointment details, test results and consultant correspondence.

A bespoke software programme, ‘DataCollector’, was built by a senior developer from the Application Development team at University Hospitals NHS Foundation Trust. DataCollector includes a ‘back end’ administrative section used to build ePROM questionnaires and display the data and a ‘front end’ which enables patients to view and complete questionnaires, and to view their longitudinal ePROM data over time, all within myHealth. DataCollector was developed using Microsoft. Net technology, mainly ASP. Net Webforms, C#, Entity framework and SQL Server. As with myHealth, all data are stored on secure servers behind the NHS trust firewall.

### User interaction

A myHealth reminder system is tasked to send monthly emails to patients asking them to complete and submit an ePROM questionnaire. However, patients are able to submit any number of additional ‘ad-hoc’ questionnaires at any time outside of the scheduled monthly reporting dates.

**Fig. 2:**
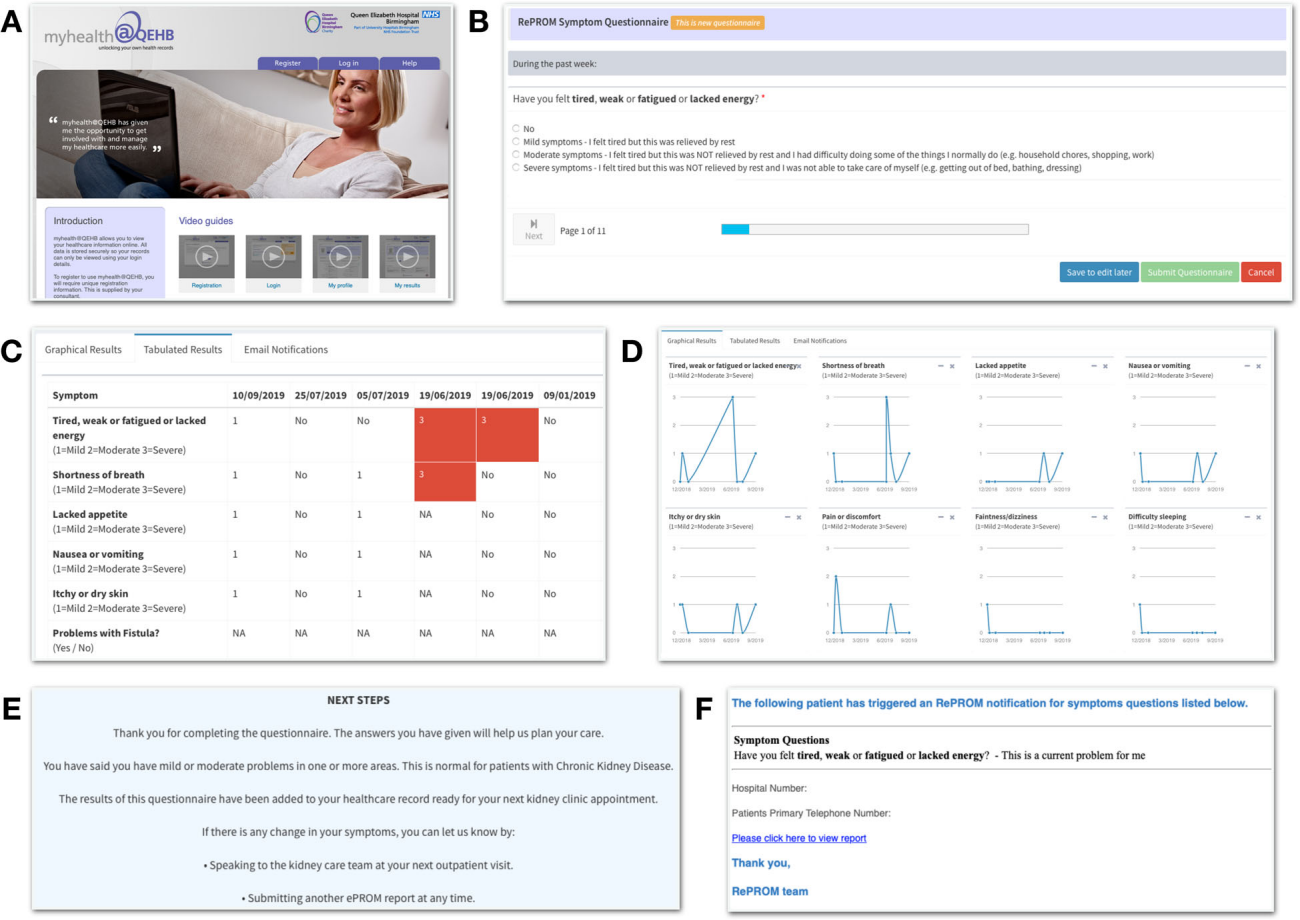
RePROM system example screenshots (dummy data). **a** myHealth home screen; **b** RePROM question page; **c** tabulated raw questionnaire data and **d** graphical display of longitudinal ePROM data, patient/clinician view identical; **e** tailored advice provided to patients upon submission of a questionnaire; **f** example of automated email notification sent to the kidney care team in response to a patient report of severe and current fatigue

After logging in to myHealth, patients are able to complete the ePROM online in approximately 6–10 min (see Fig. 2 for system screenshots). The ePROM directs patients to submit regular self-reports across a range of symptoms commonly experienced in CKD, including: fatigue; shortness of breath; loss of appetite; nausea or vomiting; itchiness or dry skin; pain; problems with fistula; faintness or dizziness; difficulty sleeping; restless legs; diarrhoea and ankle swelling. The questionnaire also includes an open text item which allows patients to report any other symptoms or problems that they would like to flag to their kidney care team.

Both patients and clinicians are able to view identical graphical and tabulated views of patient’s longitudinal ePROM report data, either remotely online (patients) or via the EPR (clinicians). This identical view was suggested by both patients and clinicians in the co-design team as an essential feature aimed at fostering a shared understanding of the patient’s symptom burden.

A system algorithm highlights severe symptoms that may require clinical follow-up, delivering pertinent information to the patient via an on screen ‘pop-up’, and sending an automated email to the kidney care team containing details of the symptom(s) triggering the notification and providing a direct link with which to access all of the patient’s ePROM reports (Fig. 1).

### Feasibility piloting and evaluation

Feasibility of the system will be evaluated as part of the National Institute for Health Research (NIHR) funded RePROM (Renal electronic Patient-Reported Outcome Measure) pilot trial (reference: PDF-2016-09-009). A protocol for the study is available [36]. The full methods and results will be published following completion.

**Fig. 3:**
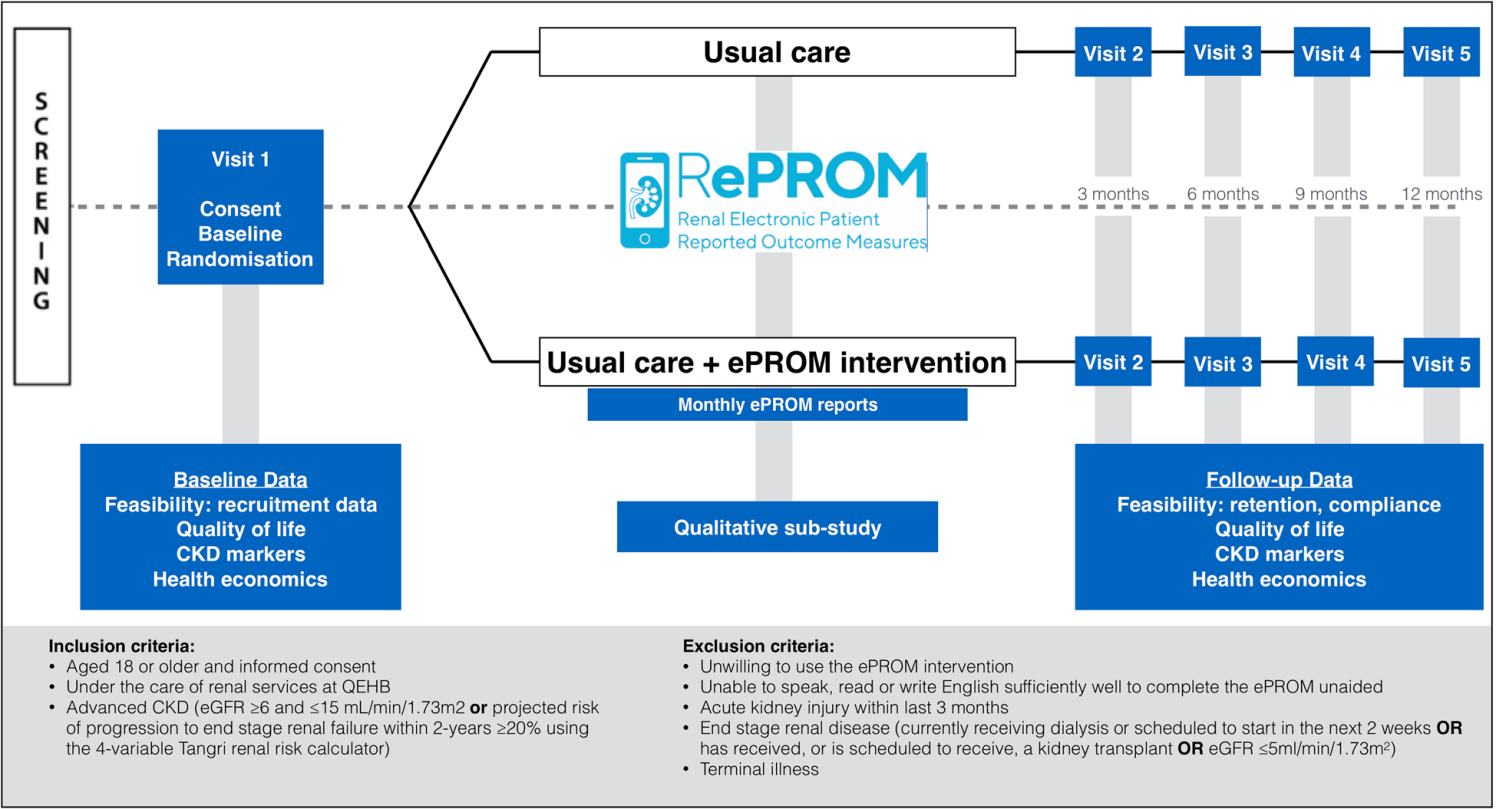
RePROM pilot trial schema

In summary, the RePROM pilot trial is an investigator led, single-centre, open-label, two-arm randomised controlled pilot trial of 66 participants aged 18 years or over with advanced CKD (trial definition: estimated Glomerular Filtration rate (eGFR) ≥6 and ≤ 15 mL/min/1.73m^2^ inclusive; or a projected risk of progression to end-stage renal failure within 2-years ≥20% using the 4-variable Tangri renal risk calculator [37]). The trial is registered with ISRCTN (ISRCTN12669006) and the NIHR Portfolio (CPMS ID: 36497). The study schema is outlined in Fig. 3.

The primary aims of the study are to pilot the trial protocol and assess the feasibility of undertaking a full-scale RCT exploring the use of an ePROM in the management of advanced CKD. This will include assessment of both quantitative and qualitative data. The pilot study will:

- Test and pilot the trial protocol (including recruitment and retention rates, data collection processes, data completeness and adherence to the ePROM intervention);
- Assess the willingness of clinicians to randomise participants into the trial;
- Assess the willingness of people with advanced CKD to be randomised into the trial;
- Assess the acceptability of the ePROM system;
- Explore the need for a non-web-based intervention platform for participants who are unable to use the online ePROM;
- Inform selection of the most appropriate primary outcome measure for the full-scale RCT;
- Provide data to help estimate the sample size for the full-scale RCT;
- Provide a platform to develop and pilot the processes to capture costs and outcomes to inform the health economic evaluation for the full-scale RCT;
- Determine key participation criteria for centre involvement in the full-scale RCT.

A range of trial outcomes will be captured 3, 6, 9 and 12-months post randomisation (assessment window +/− 3 weeks). These include clinical data e.g.: Serum Creatinine, Calcium, Phosphate, Bicarbonate, Albumin, eGFR, Albumin Creatinine Ratio, blood pressure, and for participants with diabetes: glucose and HbA1c. Patient quality of life will be assessed using a paper version of the EQ-5D-5L questionnaire, a reliable/validated generic measure of health status commonly used in CKD trials research [38]. Healthcare resource use data will also be collected, adopting a NHS/personal social care primary perspective, which will focus on healthcare resource use and costs including: renal staff activity in response to ePROM notifications; GP and hospital consultations; in-patient hospitalisation; medications; referrals; and NHS costs associated with maintenance of the ePROM system. Resource use will be valued using appropriate unit costs such as the British National Formulary and the most recent version of Unit Costs of Health and Social Care and NHS Reference Costs [39]. Integrity/performance of the ePROM system technology will be explored using both quantitative compliance data (including the proportion of expected forms returned), notification case report forms (detailing the cause of notification, clinical response and time taken to address) and qualitative debriefing interviews conducted with approximately 40 patients and clinicians/study personnel purposively selected to capture those individuals who experienced a range of outcomes and experiences during the trial where possible.

## Discussion

We have successfully developed an ePROM system which allows patients with advanced CKD to remotely self-report their symptoms using a simple and secure online platform. The system integrates symptom data into the hospital EPR and: (i) allows real-time monitoring aimed at facilitating optimal care; (ii) includes automated self-management advice for patients; and (iii) can trigger automated notification of both the patient and the clinical team in the advent of a severe symptom report.

To date, research exploring ePROM efficacy has primarily focused on cancer. Basch and colleagues conducted an RCT in the US, involving patients receiving outpatient chemotherapy for advanced solid tumours; with participants in the intervention arm asked to report 12 common symptoms on a weekly basis using the web-based STAR (Symptom Tracking and Reporting) ePROM system [12]. Four important findings emerged from this work. First, significantly more patients in the STAR arm experienced a clinically meaningful improvement in health-related quality of life at 6 months, with significantly fewer patients experiencing a clinically meaningful deterioration. Second, ePROM use was associated with fewer accident and emergency visits and inpatient hospitalisations. Third, overall survival was significantly improved in this group (median difference 5 months; median follow-up 7 years; hazard ratio 0.83, 95% CI, 0.70 to 0.99) [11]. Fourth, contact between nursing staff and patients in both arms of the study was similar, suggesting smooth integration of the ePROM system into routine practice. In addition, resources used to manage ePROM email alerts were modest, with the majority of cases resolved following telephone counselling regarding symptom management.

A survival benefit was replicated in a similar ePROM RCT, conducted by Denis et al., in a cohort of patients undergoing treatment for lung cancer in France using the ‘e-FAP’ (e-Follow-Up Application) (median survival difference 7.6 months; 2 year follow-up; hazard ratio, 0.59 95% CI, 0.37 to 0.96) [13, 14]. Again, perceived resource use was minimal, with a mean of 15 min weekly spent by the oncologist to manage all web alerts (*n* = 60 simultaneous users). Moreover, this study was the first to evaluate cost-effectiveness of an ePROM-based symptom monitoring approach (average annual cost €362 lower per patient in the ePROM arm; €20,912 total cost per Quality Adjusted Life Year) [40].

Both the STAR and e-FAP systems appeared to facilitate early detection of symptom deterioration and supportive care needs, improving patient outcomes and offering health provider/societal cost savings. In theory, such a system could lead to similar benefits in a CKD population, where effective management relies on the timely detection of clinical deterioration towards end stage kidney failure [41].

Feasibility of ePROM capture in patients with CKD has been demonstrated in Canada and the UK. Schick-Makaroff and Molzahn successfully used tablet computers to capture ePROM data from *n* = 99 patients on attendance at outpatient home dialysis clinics in two cities on the west coast of Canada over a 6-month timeframe [18]. Pittman et al. collected daily ePROM data from 43 UK patients with CKD stage 4/5 (19 receiving haemodialysis, 5 receiving peritoneal dialysis, and 19 pre-dialysis) [17]. Eighty percent submitted data for > 30 days, with 65% continuing daily submission up to 90 days. However, neither study appeared to incorporate real-time data upload to an EPR, or automated patient/clinician feedback and notifications, both key components of the STAR and e-FAP systems [12, 13].

Our system has adopted these additional real-time features and is currently being evaluated in the RePROM pilot trial in a pre-dialysis population. We intend to explore the experiences of patients using RePROM, compared to those documented using the aforementioned ePROM systems in renal disease [17, 18] and cancer [12, 13]. Assuming feasibility is demonstrated, and after any necessary refinements, the final RePROM system will be included in a planned multi-centre RCT.

### Limitations

As mentioned, our co-design group were unable to endorse an existing, validated, PROM for use within our system as planned. Systematic review demonstrated that renal PROMs frequently lacked validation in English-speaking populations (particularly in the UK) and were missing evidence to support important measurement properties including measurement error, structural validity, responsiveness and patient acceptability [29]. There is a need to develop a renal ePROM that minimises questionnaire burden whilst optimising precision and which demonstrates appropriate validity, reliability, responsiveness and acceptability to be used at an individual patient level in the NHS. Thus, we are currently in the process of developing and validating a formal renal symptom item bank, computerised adaptive testing system and paper-based short-form (Kidney Research UK funding, ref.: KS_RP_013_20180914), which will be incorporated into the existing ePROM system prior to evaluation in a RCT. Whilst incorporation of the ePROM within a pre-existing hospital patient portal allowed us to take advantage of inbuilt security features, constraints within the portal system meant we were unable to implement a telephone-based interactive voice recognition feature to support patients with limited online access, as we had initially planned. In addition, at the time of implementation, the portal was not optimised for smartphone usage, a potential barrier to access that we intend to explore in the process evaluation phase of the feasibility study.

## Conclusions

Routine ePROM collection and real-time feedback in patients with advanced CKD has the potential to improve outcomes and reduce health service costs. We have successfully developed a trial-ready ePROM system, the feasibility of which is currently being explored in a pilot study. An updated system, incorporating a validated renal symptom item bank currently in development, will be subjected to evaluation in a future UK-based multi-centre RCT.

## Data Availability

The datasets used and/or analysed during the current study are available from the corresponding author on reasonable request.

## Abbreviations

CKD: Chronic kidney disease
ePROMs: Electronic patient-reported outcome measures
EPR: Electronic patient record
RCT: Randomised controlled trial
QEHB: Queen Elizabeth Hospital Birmingham
NHS: National Health Service
UHBFT: University Hospitals Birmingham Foundation Trust
NIHR: National Institute for Health Research

## References

[1] Jager KJ, Fraser SDS (2017). The ascending rank of chronic kidney disease in the global burden of disease study. Nephrol Dial Transplant.

[2] Almutary H, Bonner A, Douglas C (2013). Symptom burden in chronic kidney disease: a review of recent literature. J Renal Care.

[3] TKHA G. Kidney health: delivering excellence. Kidney Research UK. 2013; [Available at: http://www.kidneyresearchuk.org/file/media/Kidney-Health-Delivering-Excellence-1709-15 Oct.pdf Accessed Nov 2015].

[4] Jesky MD, Dutton M, Dasgupta I, Yadav P, Ng KP, Fenton A (2016). Health-related quality of life impacts mortality but not progression to end-stage renal disease in pre-dialysis chronic kidney disease: a prospective observational study. PLoS One.

[5] Calvert M, Kyte D, Price G, Valderas JM, Hjollund NH (2019). Maximising the impact of patient reported outcome assessment for patients and society. BMJ..

[6] Holch P, Warrington L, Bamforth L, Keding A, Ziegler L, Absolom K (2017). Development of an integrated electronic platform for patient self-report and management of adverse events during cancer treatment. Ann Oncol.

[7] Velikova G, Brown JM, Smith AB, Selby PJ (2002). Computer-based quality of life questionnaires may contribute to doctor-patient interactions in oncology. Br J Cancer.

[8] Detmar SB, Muller MJ, Schornagel JH, Wever LD, Aaronson NK (2002). Health-related quality-of-life assessments and patient-physician communication: a randomized controlled trial. JAMA..

[9] McCann L, Maguire R, Miller M, Kearney N (2009). Patients’ perceptions and experiences of using a mobile phone-based advanced symptom management system (ASyMS©) to monitor and manage chemotherapy related toxicity. Eur J Cancer Care.

[10] Velikova G, Booth L, Smith AB, Brown PM, Lynch P, Brown JM (2004). Measuring quality of life in routine oncology practice improves communication and patient well-being: a randomized controlled trial. J Clin Oncol.

[11] Basch E, Deal AM, Dueck AC, Scher HI, Kris MG, Hudis C (2017). Overall survival results of a trial assessing patient-reported outcomes for symptom monitoring during routine cancer treatment. JAMA..

[12] Basch, E., Deal, A., Kris, M., Scher, H., Hudis, C., Sabbatini, P., et al. (2015). Symptom monitoring with patient-reported outcomes during routine cancer treatment: a randomized controlled trial. *J Clin Oncol*. 10.1200/JCO.2015.63.0830. 10.1200/JCO.2015.63.0830 PMC4872028 26644527

[13] Denis, F., Lethrosne, C., Pourel, N., Molinier, O., Pointreau, Y., Domont, J., et al. (2017). Randomized trial comparing a web-mediated follow-up with routine surveillance in lung cancer patients. *J Natl Cancer Inst, 109*(9). 10.1093/jnci/djx029. 10.1093/jnci/djx029 28423407

[14] Denis F, Basch E, Septans A-L, Bennouna J, Urban T, Dueck AC (2019). Two-year survival comparing web-based symptom monitoring vs routine surveillance following treatment for lung cancer. JAMA..

[15] Hays RD, Kallich JD, Mapes DL, Coons SJ, Amin N, Carter WB (1997). Kidney Disease Quality of Life Short Form (KDQOL-SF), Version 1.3: a manual for use and scoring.

[16] Hays RD, Kallich JD, Mapes DL, Coons SJ, Carter WB (1994). Development of the kidney disease quality of life (KDQOL) instrument. Qual Life Res.

[17] Pittman ZC, John SG, McIntyre CW (2017). Collection of daily patient reported outcomes is feasible and demonstrates differential patient experience in chronic kidney disease. Hemodial Int.

[18] Schick-Makaroff K, Molzahn AE (2017). Evaluation of real-time use of electronic patient-reported outcome data by nurses with patients in home dialysis clinics. BMC Health Serv Res.

[19] Möhler R, Köpke S, Meyer G (2015). Criteria for reporting the development and evaluation of complex interventions in healthcare: revised guideline (CReDECI 2). Trials..

[20] Greenhalgh J, Dalkin S, Gooding K, Gibbons E, Wright J, Meads D (2017). Functionality and feedback: a realist synthesis of the collation, interpretation and utilisation of patient-reported outcome measures data to improve patient care. Health Serv Deliv Res.

[21] Greenhalgh J (2009). The applications of PROs in clinical practice: what are they, do they work, and why?. Qual Life Res.

[22] Greenhalgh J, Long AF, Flynn R (2005). The use of patient reported outcome measures in routine clinical practice: lack of impact or lack of theory?. Soc Sci Med.

[23] Santana M-J, Feeny D (2014). Framework to assess the effects of using patient-reported outcome measures in chronic care management. Qual Life Res.

[24] Feldman-Stewart D, Brundage MD (2008). A conceptual framework for patient–provider communication: a tool in the PRO research tool box. Qual Life Res.

[25] Snyder CF, Aaronson NK, Choucair AK, Elliott TE, Greenhalgh J, Halyard MY (2012). Implementing patient-reported outcomes assessment in clinical practice: a review of the options and considerations. Qual Life Res.

[26] Etkind SN, Daveson BA, Kwok W, Witt J, Bausewein C, Higginson IJ (2015). Capture, transfer, and feedback of patient-centered outcomes data in palliative care populations: does it make a difference? A systematic review. J Pain Symptom Manag.

[27] Street RL, Makoul G, Arora NK, Epstein RM (2009). How does communication heal? Pathways linking clinician–patient communication to health outcomes. Patient Educ Couns.

[28] Warrington L, Absolom K, Velikova G (2015). Integrated care pathways for cancer survivors–a role for patient-reported outcome measures and health informatics. Acta Oncol.

[29] Aiyegbusi OL, Kyte D, Cockwell P, Marshall T, Gheorghe A, Keeley T (2017). Measurement properties of patient-reported outcome measures (PROMs) used in adult patients with chronic kidney disease: a systematic review. PLoS One.

[30] Agarwal R (2010). Developing a self-administered CKD symptom assessment instrument. Nephrol Dial Transplant.

[31] Chao S, Yen M, Lin T-C, Sung J-M, Wang M-C, Hung S-Y (2016). Psychometric properties of the kidney disease quality of life–36 questionnaire (KDQOL-36™). West J Nurs Res.

[32] Ricardo AC, Hacker E, Lora CM, Ackerson L, DeSalvo KB, Go A (2013). Validation of the kidney disease quality of life short form 36 (KDQOL-36™) US Spanish and English versions in a cohort of hispanics with chronic kidney disease. Ethn Dis.

[33] Cheung YB, Seow YY, Qu LM, Yee ACP (2012). Measurement properties of the Chinese version of the kidney disease quality of life-short form (KDQOL-SF™) in end-stage renal disease patients with poor prognosis in Singapore. J Pain Symptom Manag.

[34] ElHafeez SA, Sallam SA, Gad ZM, Zoccali C, Torino C, Tripepi G (2012). Cultural adaptation and validation of the “Kidney Disease and Quality of Life-Short Form (KDQOL-SF™) version 1.3” questionnaire in Egypt. BMC Nephrol.

[35] Aiyegbusi OL, Kyte D, Cockwell P, Marshall T, Dutton M, Walmsley-Allen N (2018). Development and usability testing of an electronic patient-reported outcome measure (ePROM) system for patients with advanced chronic kidney disease. Comput Biol Med.

[36] Kyte D, Bishop J, Brettell E, Calvert M, Cockwell P, Dutton M (2018). Use of an electronic patient-reported outcome measure in the management of patients with advanced chronic kidney disease: the RePROM pilot trial protocol. BMJ Open.

[37] Tangri N, Stevens LA, Griffith J, Tighiouart H, Djurdjev O, Naimark D (2011). A predictive model for progression of chronic kidney disease to kidney failure. JAMA..

[38] Devlin NJ, Krabbe PF (2013). The development of new research methods for the valuation of EQ-5D-5L. Eur J Health Econ.

[39] Curtis L (2015). Unit costs of health and social care 2015. Personal social services research unit.

[40] Lizée T, Basch E, Trémolières P, Voog E, Domont J, Peyraga G (2019). Cost-effectiveness of web-based patient-reported outcome surveillance in patients with lung cancer. J Thorac Oncol.

[41] Braun L, Sood V, Hogue S, Lieberman B, Copley-Merriman C (2012). High burden and unmet patient needs in chronic kidney disease. Int J Nephrol Renov Dis.

